# 1128. Efficacy and Safety of Nelfinavir in Asymptomatic and Mild COVID-19 Patients: A Multicenter, Randomized Controlled Trial

**DOI:** 10.1093/ofid/ofac492.967

**Published:** 2022-12-15

**Authors:** Shigeru Kohno, Taiga Miyazaki, Naoki Hosogaya, Shinpei Morimoto, Hiroshi Yamamoto, Shingo Iwami, Yoshitsugu Miyazaki, Hideki Hanaoka

**Affiliations:** Nagasaki University, Nagasaki, Nagasaki, Japan; University of Miyazaki, Miyazaki, Miyazaki, Japan; Nagasaki University, Nagasaki, Nagasaki, Japan; Nagasaki University, Nagasaki, Nagasaki, Japan; Nagasaki University, Nagasaki, Nagasaki, Japan; Nagoya University, Nagoya, Aichi, Japan; National Institute of Infectious Diseases, Shinjuku-ku, Tokyo, Japan; Chiba University, Chiba, Chiba, Japan

## Abstract

**Background:**

Nelfinavir, an orally administered inhibitor of human immunodeficiency virus protease, inhibited the replication of severe acute respiratory syndrome coronavirus 2 (SARS-CoV-2) *in vitro*. To evaluate the efficacy and safety of nelfinavir, we conducted a randomized controlled trial.

**Methods:**

Adult patients testing positive for SARS-CoV-2 infection within 3 days were eligible for the study if they had no or mild symptoms of coronavirus disease 2019. Exclusion criteria included the followings: onset of symptoms ≥ 8 days before enrollment; oxygen saturation of 95% or less on room air; and vaccinated patients. Patients were randomly assigned (1:1) to receive oral nelfinavir 750 mg (×3 times daily) combined with standard-of-care or standard-of-care alone. The primary endpoint was the time to clearance of SARS-CoV-2. Saliva was collected every day and viral load was measured by quantitative reverse transcription-polymerase chain reaction (qRT-PCR). Nelfinavir was administered for 14 days. However, the treatment could be discontinued by the decision of investigator, if patients had 2 consecutive negative test results by qRT-PCR. Clinical course and safety information were collected through day 28. The study is registered with the Japan Registry of Clinical Trials (number, jRCT2071200023).

**Results:**

Between July 2020 and October 2021, 123 patients (63 in the nelfinavir group and 60 in the control group) were enrolled into the study and included in the analysis. The median time to viral clearance was 8.0 (95% confidence interval [CI] 7.0 to 12.0) days in the nelfinavir group and 8.0 (95% CI 7.0 to 10.0) days in the control group without statistically significant difference between the treatment group (hazard ratio 0.815, 95% CI 0.563 to 1.182; *P* = 0.1870).

Adverse events were reported in 47 (74.6%) patients in the nelfinavir group and 20 (33.3%) in the control group. The most common adverse events in the nelfinavir group were diarrhea (49.2%) and nausea (6.3%).

**Conclusion:**

Nelfinavir did not reduce the time to viral clearance in this setting.

**Disclosures:**

**Shigeru Kohno, n/a**, Pfizer: Grant/Research Support|Pfizer: Honoraria **Taiga Miyazaki, n/a**, Pfizer: Advisor/Consultant|Pfizer: Grant/Research Support|Pfizer: Honoraria.

